# Molecular and Functional Effects of a Splice Site Mutation in the *MYL2* Gene Associated with Cardioskeletal Myopathy and Early Cardiac Death in Infants

**DOI:** 10.3389/fphys.2016.00240

**Published:** 2016-06-17

**Authors:** Zhiqun Zhou, Wenrui Huang, Jingsheng Liang, Danuta Szczesna-Cordary

**Affiliations:** Department of Molecular and Cellular Pharmacology, University of Miami Leonard M. Miller School of MedicineMiami, FL, USA

**Keywords:** cardioskeletal myopathy, actin-myosin interaction, fluorescence measurements, myosin ATPase, muscle contraction

## Abstract

The homozygous appearance of the intronic mutation (IVS6-1) in the *MYL2* gene encoding for myosin ventricular/slow-twitch skeletal regulatory light chain (RLC) was recently linked to the development of slow skeletal muscle fiber type I hypotrophy and early cardiac death. The IVS6-1 (c403-1G>C) mutation resulted from a cryptic splice site in *MYL2* causing a frameshift and replacement of the last 32 codons by 19 different amino acids in the RLC mutant protein. Infants who were IVS6-1^+∕+^-positive died between 4 and 6 months of age due to cardiomyopathy and heart failure. In this report we have investigated the molecular mechanism and functional consequences associated with the IVS6-1 mutation using recombinant human cardiac IVS6-1 and wild-type (WT) RLC proteins. Recombinant proteins were reconstituted into RLC-depleted porcine cardiac muscle preparations and subjected to enzymatic and functional assays. IVS6-1-RLC showed decreased binding to the myosin heavy chain (MHC) compared with WT, and IVS6-1-reconstituted myosin displayed reduced binding to actin in rigor. The IVS6-1 myosin demonstrated a significantly lower V_max_ of the actin-activated myosin ATPase activity compared with WT. In stopped-flow experiments, IVS6-1 myosin showed slower kinetics of the ATP induced dissociation of the acto-myosin complex and a significantly reduced slope of the k_obs_-[MgATP] relationship compared to WT. In skinned porcine cardiac muscles, RLC-depleted and IVS6-1 reconstituted muscle strips displayed a significant decrease in maximal contractile force and a significantly increased Ca^2+^ sensitivity, both hallmarks of hypertrophic cardiomyopathy-associated mutations in *MYL2*. Our results showed that the amino-acid changes in IVS6-1 were sufficient to impose significant conformational alterations in the RLC protein and trigger a series of abnormal protein-protein interactions in the cardiac muscle sarcomere. Notably, the mutation disrupted the RLC-MHC interaction and the steady-state and kinetics of the acto-myosin interaction. Specifically, slower myosin cross-bridge turnover rates and slower second-order MgATP binding rates of acto-myosin interactions were observed in IVS6-1 vs. WT reconstituted cardiac preparations. Our *in vitro* results suggest that when placed *in vivo*, IVS6-1 may lead to cardiomyopathy and early death of homozygous infants by severely compromising the ability of myosin to develop contractile force and maintain normal systolic and diastolic cardiac function.

## Introduction

**Figure 1:**
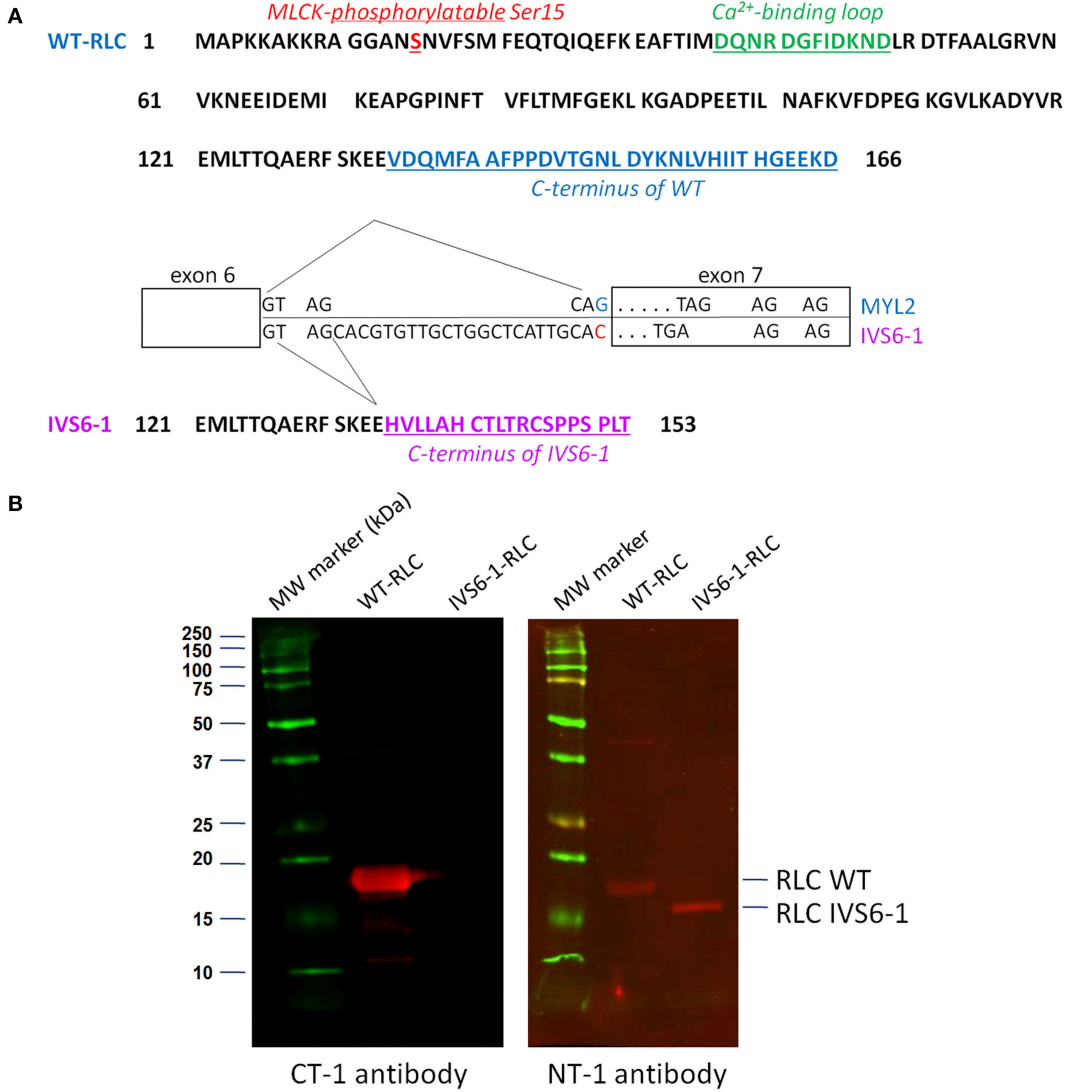
**(A)** Amino acid sequences of myosin RLC WT and IVS6-1 mutant protein. IVS6-1 is a splice site mutation 1 (c403-1G>C) that occurs in Intron 6 of the *MYL2* gene encoding for the human ventricular RLC/ slow-twitch skeletal muscle RLC. As a consequence, the last 32 amino acids of WT (blue) are replaced by 19 different amino acids (purple) in IVS6-1. The phosphorylation site at Ser15 is shown in red and the RLC calcium binding loop in green. The exon-intron sequence is modified from Weterman et al. (2013). **(B)** Western blots of recombinant human ventricular RLC WT and RLC IVS6-1 mutant detected with the C-terminal RLC antibody (CT-1) and the N-terminal RLC antibody (NT-1). Note the loss of C-terminal epitope in IVS6-1 enabling the mutant to be exclusively detected with NT-1 compared with RLC-WT, detected with both, CT-1 and NT-1 antibodies.

A new skeletal muscle fiber type-I myopathy with progressive cardiomyopathy and the early death of infants due to cardiac failure was reported in three unrelated Dutch families by Barth et al. (1998). It was not until recently that the genetic cause of this cardioskeletal disorder was identified by Weterman et al. (2013), and related to mutations in the *MYL2* gene encoding for the ventricular and slow-twitch skeletal myosin regulatory light chain (RLC). To date, about 16 single amino acid mutations in *MYL2* have been linked to various forms of cardiomyopathy (Poetter et al., 1996; Flavigny et al., 1998; Andersen et al., 2001, 2009; Kabaeva et al., 2002; Richard et al., 2003; Olivotto et al., 2008; Garcia-Pavia et al., 2011; Claes et al., 2015; Huang et al., 2015). The IVS6-1 (c403-1G>C) mutation, associated with the slow-skeletal and cardiac muscle myopathy, resulted from a cryptic splice site upstream of the last exon of *MYL2* causing a frameshift and replacement of the last 32 codons by 19 different codons (Weterman et al., 2013). As a consequence, the C-tail of the RLC protein was truncated and contained a completely altered C-terminal amino acid sequence compared with wild-type (WT) RLC (NCBI accession # P10916) (Figure 1A). Immunohistochemical staining of skeletal muscle tissue of the Dutch patients homozygous for IVS6-1 showed a diffuse and weak expression of the mutant protein without clear fiber specificity, while the normal RLC protein was absent (Weterman et al., 2013). Therefore, in this report we aimed at elucidating the potential molecular mechanism by which the IVS6-1 mutation may exert its effects on cardiac muscle contraction. This process is highly dependent upon the integrity of myosin, including its two heavy chains (MHC) and the regulatory and essential (ELC) light chains (Holmes and Geeves, 2000), and such drastic amino acid changes in the sequence of RLC due to IVS6-1 are likely to affect the interaction of myosin with actin, force production and lead to cardiac dysfunction responsible for infantile death of IVS6-1 homozygous patients (Barth et al., 1998; Weterman et al., 2013).

Previous studies from our lab demonstrated that single amino acid mutations in the RLC protein shown to be associated with hypertrophic cardiomyopathy (HCM), were able to cause significant changes in the secondary structure of RLC, as well as in the Ca^2+^ binding and phosphorylation properties (Szczesna et al., 2001). They also adversely affected the function of mutated myosin and its ability to interact with actin and produce contractile force (Szczesna-Cordary et al., 2004; Greenberg et al., 2010; Farman et al., 2014; Muthu et al., 2014; Karabina et al., 2015). In this report we have examined, for the first time, the effect of the IVS6-1 mutation on the molecular rearrangements in the RLC and the function of mutant myosin *in vitro*. The interactions of IVS6-1 with the MHC, and the mutant myosin with actin were investigated using recombinant human cardiac RLC proteins, IVS6-1 vs. WT, that could be reconstituted into porcine cardiac muscle preparations, myosin and skinned muscle strips. Prior to reconstitution, the preparations were stripped of endogenous porcine cardiac RLC. We show that in a way similar to that of other HCM causing mutations in myosin RLC, the IVS6-1 mutation induced significant changes in the RLC structure and the function of mutant myosin compromising its interaction with actin and ultimately leading to dysregulated cardiac muscle contraction.

## Materials and methods

### Cloning, expression and purification of wild-type (WT) human cardiac RLC and the IVS6-1 mutant

The RLC WT was cloned, expressed and purified as previously described (Szczesna et al., 2001). The cDNA of IVS6-1 was synthesized and inserted in the pCR2.1 vector by Eurofins MWG Operon™. The plasmid was amplified in Subcloning Efficiency™ DH5α™ competent cells (Invitrogen) and was transformed into BL21(DE3) competent cells (Agilent Technologies) for expression. Similar to WT, the IVS6-1 mutant protein was purified using an S-Sepharose column followed by a Q-Sepharose column chromatography (GE Healthcare Life Science). The S-Sepharose column was equilibrated with 6 M urea, 20 mM Citrate, 0.1 mM phenylmethylsulfonyl fluoride (PMSF), 1 mM dithiothreitol (DTT), 0.02% NaN_3_, pH 6.0. Proteins were eluted using 800 ml salt gradient of 0−450 mM NaCl. For Q-Sepharose purification the following buffer was used: 3 M urea, 25 mM Tris-HCl, pH 7.5, 0.1 mM PMSF, 1 mM DTT, and 0.02% NaN_3_, and proteins were eluted with a 1000 ml salt gradient of 0–450 mM KCl. The final purity of the proteins was assessed by 15% SDS-PAGE. The N-terminal peptide RLC antibody (NT-1 RLC, anti-rabbit, aa 7–21) and the C-terminal peptide RLC antibody (CT-1, anti-rabbit, aa 143–156), both produced in this laboratory (Wang et al., 2006), were used to verify the quality and purity of IVS6-1 and WT proteins. Proteins were stored in Q-Sepharose buffer at −80°C until used in experiments.

### Myosin light chain kinase (MLCK)-dependent phosphorylation of IVS6-1 vs. WT RLCs

RLC WT and IVS6-1 proteins were first dialyzed into phosphorylation buffer containing 30 mM KCl, 20 mM PO_4_, pH 8.0, and adjusted to a concentration of 1.5 mg/ml. Skeletal muscle MLCK was prepared as described previously (Greenberg et al., 2009). Phosphorylation reaction was performed with 5.5 μM MLCK, 5 μM calmodulin (CaM), 0.1 mM CaCl_2_, 12.5 mM MgCl_2_, and 5 mM ATP for 30 min at room temperature. The reaction was terminated by adding 8 M urea. The degree of phosphorylation was assessed by 8% PAGE. The samples were prepared by mixing 100 μl of protein with 70 mg of ultrapure urea, 10 μl β-ME (β-mercaptoethanol), and 5 μl of Bromophenol Blue. 8 M urea–containing gels were run at 100 Volts for 180 min. Gels were scanned and analyzed using Image J software.

### Preparation of porcine cardiac (PC) myosin

Left ventricular (LV) muscles of pig hearts obtained postmortem from a slaughterhouse, chilled on ice and washed clear of blood with ice-cold H_2_O were isolated and minced. The muscle mince was rinsed with ice-cold H_2_O until clear followed by extraction of myosin using Edsall–Weber solution (0.012 M Na_2_CO_3_, 0.04 M NaHCO_3_, and 0.6 M KCl, pH 9.0; 300 ml/100 g of muscle) on ice with stirring for 1.5 h, as described earlier in Pant et al. (2009). The homogenate was then centrifuged at 13,000 g for 20 min, and the supernatant was precipitated with 13 vol of ice-cold water containing 1 mM EDTA (ethylenediaminetetraacetic acid) and 1 mM DTT, followed by centrifugation at 13,000 g for 10 min. The pellet was resuspended in buffer containing 0.5 M KCl, 20 mM MOPS (pH 7.0), 1 mM DTT, and 10 mM MgATP and centrifuged at 186,000 g for 1.5 h. Supernatant containing native PC myosin was precipitated with 14 vol of ice-cold H_2_O containing 1 mM DTT and centrifuged at 8000 g for 10 min. The pellet (kept on ice overnight) was resuspended in 0.5 M KCl, 20 mM MOPS (pH 7.0), 1 mM DTT, and 10 mM MgATP and centrifuged at 186,000 g for 1.5 h. The supernatant containing PC myosin was tested for purity by SDS-PAGE, mixed with glycerol (1:1 vol/vol), and stored at −20°C until needed.

### Depletion of endogenous RLC from PC myosin and reconstitution of myosin with RLC WT and IVS6-1

About 1.5 mg/ml of PC myosin dissolved in 0.5 M KCl and 10 mM Potassium Phosphate (pH 8.5) was incubated in buffer containing 1% Triton X-100 and 5 mM CDTA (1,2-cyclohexylenedinitrilotetraacetic) for 30 min at room temperature to extract endogenous PC-RLC. Then the mixture was precipitated with 13 vol of ice-cold water containing 1 mM DTT for 30 min on ice and centrifuged at 8000 g for 10 min. The pellet containing RLC-depleted PC myosin was re-suspended in reconstitution buffer (0.4 M KCl, 50 mM MOPS, pH 7.0, 2 mM MgCl_2_, and 1 mM DTT) to ~2.8 μM concentration and titrated with increasing concentrations of human cardiac WT or IVS6-1 RLCs (from 0.1 to 14 μM). The molar ratio of RLC to depleted myosin ranged from 0.1 to 5.0. Titrations were performed in the presence of BSA to prevent nonspecific RLC binding. The mixtures were incubated for 30 min at room temperature and then precipitated with 13 vol of ice-cold water containing 1 mM DTT for 30 min on ice, and centrifuged at 8000 g for 10 min at 4°C. The pellets containing WT or IVS6-1 -reconstituted myosins were dissolved in small volumes of 3 M KCl to reach a final concentration of 0.5 M KCl, and clarified by ultracentrifugation at 200,000 g for 45 min at 4°C. Resulting samples were examined by SDS-PAGE. Gel bands were scanned and quantified using Image J software. The degree of reconstitution was calculated based upon the RLC/ELC band intensity ratio of native, RLC-depleted and WT/IVS6-1-reconstituted PC myosin with ELC bands used as loading controls (Pant et al., 2009). To account for the amount of porcine RLC remaining in RLC-depleted myosin, the RLC/ELC ratio of RLC-depleted myosin was subtracted from the RLC/ELC ratio of WT-reconstituted myosin due to the similar migration pattern of the endogenous porcine RLC and exogenous human RLC. For IVS6-1, the band of IVS6-1 migrates independently of porcine RLC because of different molecular weights of the two proteins, and as such, could be assessed directly. The resultant RLC/ELC ratio in WT/IVS6-1 reconstituted myosin was then divided by the RLC/ELC ratio measured in native PC myosin. The binding isotherms were fitted to the ligand binding equation:

(1) $$\begin{array}{r} f=y_{0}\;+\;Bmax\;*\;x∕\left(Kd\;+\;x\right) \end{array}$$

where “*B*_*max*_” depicts maximal RLC binding and *K*_*d*_ is apparent dissociation constant.

Preparation of WT or IVS6-1 reconstituted myosins for the *in vitro* steady state and kinetics experiments—The RLC-depleted porcine myosin, obtained as described above, was incubated with a 3 molar excess of human recombinant WT/IVS6-1 RLCs and the mixtures were dialyzed for 2 h at 4°C against the reconstitution buffer containing 0.4 M KCl, 50 mM MOPS, pH 7.0, 2 mM MgCl_2_, and 1 mM DTT. The protein complexes were centrifuged at 8000 g for 10 min and then dialyzed overnight at 4°C against 5 mM DTT to precipitate the RLC-reconstituted myosin. The samples were then centrifuged at 8000 g for 10 min to collect the reconstituted myosins and the pellets resuspended in 0.4 M KCl, 10 mM MOPS, pH 7.0, 1 mM DTT. This procedure yielded fully reconstituted IVS6-1- and WT-myosins.

### Preparation of actin and labeling with pyrene iodoacetamide

Rabbit skeletal acetone powder was extracted with a G-actin buffer consisting of 2 mM Tris-HCl (pH 8), 0.2 mM Na_2_ATP, 0.5 mM β-ME, 0.2 mM CaCl_2_, and 0.0005% NaN_3_ at a ratio of 20 ml g^−1^ for 30 min with stirring on ice (Pardee and Spudich, 1982). The extract was clarified by filtration through several layers of cheesecloth, then centrifuged at 11,000 g at 4°C for 1 h and the tissue pellet was discarded. The supernatant was adjusted to a final concentration of 50 mM KCl, 2 mM MgCl_2_, and 1 mM Na_2_ATP (pH 8.0) and the F-actin was allowed to polymerize for 2 h at 4°C. The KCl concentration was then increased very slowly to a final concentration of 0.6 M and the mixture was stirred slowly on ice for 30 min to remove possible traces of tropomyosin–troponin (Tm–Tn). F-actin pellet was then collected by ultracentrifugation at 160,000 g at 4°C for 1.5 h. F-actin pellet was re-dissolved in a buffer containing of 10 mM MOPS (pH 7.0) and 40 mM KCl for pyrene labeling. F-actin at a concentration of 20–40 μM was incubated at room temperature in the dark, for 16 h, with a 10 molar excess of Pyrene Iodoacetamide (PIA) (Invitrogen/Molecular Probes) in F-actin buffer containing 10 mM MOPS (pH 7.0) and 40 mm KCl as previously described by Cooper et al. (1983), Kazmierczak et al. (2009). The reaction was quenched by adding 1 mM DTT and the preparation was centrifuged at 1000 g for 1 h to clarify the F-actin solution and remove precipitated PIA. F-actin was then dialyzed against 2 mM Tris–HCl, pH 8.0, 0.2 mM CaCl_2_, 0.2 mM ATP, and 1 mM DTT overnight to depolymerize F-actin and remove excess PIA. G-actin was then polymerized into F-actin overnight at 4°C by dialysis in 40 mM KCl, 1 mM MgCl_2_, and 10 mM MOPS, pH 7. Pyrene-labeled F-actin was tested spectroscopically to determine efficiency of labeling using the molar extinction coefficient, e_344_(pyrene) = 22,000 M^−1^cm^−1^. The usual molar ratio of pyrene/F-actin was ~0.8 (Kazmierczak et al., 2012).

### Fluorescence based actin-myosin binding assays

RLC WT/IVS6-1 reconstituted myosin was added at 0.05 μM increments to pyrene labeled F-actin (0.5 μM) until reaching ~2 fold molar excess over the concentration of actin. Fluorescence measurements were carried using a JASCO 6500 Spectrofluorometer. PIA was excited at 340 nm and fluorescence was collected at 407 nm. The titration data were fitted to the following quadratic equation to obtain the binding constant (K_d_) and stoichiometry (n):

(2) $$\begin{array}{l} \;f=m_{1}-m_{2}(K_{d}+n\;*\;a+x\;-\\ \sqrt{\left({\left(K_{d}+n*a+x\right)}^{2}-4*n*a*x\right)})/(2*n*a) \end{array}$$

Where *m*_1_ = initial signal, *m*_2_ = maximal amplitude (decrease in fluorescence intensity on myosin binding to pyrene–actin), n = stoichiometry of myosin-actin binding, a = concentration of actin and *x* = total concentration of added myosin.

### Stopped-flow kinetic measurements

Reconstituted myosin at a concentration of 0.25 μM were mixed with 0.25 μM pyrene labeled F-actin (stabilized by 0.25 μM phalloidin) in rigor buffer containing 0.4 M KCl, 1 mM DTT and 10 mM MOPS, pH 7.0. The complexes were mixed in a 1:1 (vol/vol) ratio with increasing concentrations of MgATP (10–150 μM) dissolved in the same buffer in the stopped flow apparatus. The time course of the change in pyrene fluorescence on MgATP-dependent myosin dissociation from actin was monitored. Measurements were performed using a BioLogic (Claix, France) model SFM-20 stopped-flow instrument outfitted with a Berger ball mixer and an FC-8 observation cuvette. The data were collected and digitized using a JASCO 6500 Fluorometer. The estimated dead time was 3.5 ms. The pyrene-F actin was excited at 347 nm and emission was monitored at 404 nm using monochromators set to 20-nm bandwidths. Typically, 8–12 stopped-flow records were averaged and fit to an exponential equation to obtain the rate at a given MgATP concentration. A plot of the observed myosin dissociation rates as a function of [MgATP] was linear and the slope corresponded to the rate constant expressed in M^−1^^*^ s^−1^.

### Myosin ATPase activity assay

Actin-activated myosin ATPase activity assays were performed in a 120 μl reaction volume in a buffer containing 25 mM imidazole, pH 7.0, 4 mM MgCl_2_, 1 mM EGTA, and 1 mM DTT and the final KCl concentration of 107 mM, as described in Kazmierczak et al. (2012). Briefly, ~1.9 μM myosin dissolved in 0.4 M KCl (in monomeric form) was added to the 96-well microplate containing increasing concentrations of F-actin (in μM): 0.1, 1, 2.5, 5, 7.5, 10, 15, 20, and 25. Protein mixtures were first incubated on ice for 10 min and then for another 10 min at 30°C. The reactions (run in triplicate) were initiated with the addition of 2.5 mM ATP with mixing in a Jitterbug incubator shaker (Boekel), allowed to proceed for 20 min at 30°C and then terminated by the addition of 30 μl 20% trichloroacetic acid (TCA). Precipitated proteins were cleared by centrifugation at 4000 g for 15 min and the inorganic phosphate was determined using the Fiske Subbarow method (Fiske and Subbarow, 1925). Data were analyzed using the Michaelis–Menten equation yielding the V_max_ and K_m_ parameters (Trybus, 2000).

### CDTA-extraction of endogenous RLC from skinned porcine papillary muscle strips and reconstitution with WT and IVS6-1

Freshly isolated porcine hearts were placed in oxygenated physiological salt solution of 140 mM NaCl, 4 mM KCl, 1.8 mM CaCl_2_, 1.0 mM MgCl_2_, 1.8 mM NaH_2_PO_4_, 5.5 mM glucose, and 50 mM Hepes buffer, pH 7.4. The papillary muscles of the left ventricles were isolated, dissected into muscle bundles of about 20 mm (length) × 3 mm (diameter), and chemically skinned in a 50% glycerol, 50% pCa 8 buffer (10^−8^ M [Ca^2+^], 1 mM free [Mg^2+^] (total MgPr -propionate = 3.88 mM), 7 mM EGTA, 2.5 mM [Mg-ATP^2−^], 20 mM MOPS, pH 7.0, 15 mM creatine phosphate and 15 units/ml of phosphocreatine kinase, ionic strength = 150 mM adjusted with KPr containing 1% Triton X-100 for 24 h at 4°C (Muthu et al., 2014). Then the strips were transferred to the same solution without Triton X-100 and stored at −20°C. Depletion of endogenous RLC from porcine cardiac muscle preparations was achieved in strips about 1.4 mm long and 100 μm wide, isolated from glycerinated papillary muscle bundles in buffer containing 5 mM CDTA, 40 mM Tris, 50 mM KCl, 1 μg/ml pepstatin A, 0.6 mM NaN_3_, 0.2 mM PMSF, and 1% Triton X-100, pH 8.4 and protease inhibitor cocktail for 65 min at room temparature. After depletion, the fibers were washed in pCa 8 solution, incubated with 40 μM RLC-WT or IVS6-1 protein and 2 mM DTT for total 40 min with fresh proteins added after 20 min. Due to a potential partial loss of TnC that can occur during extraction of RLC and to assure fibers' functionality, both the RLC and TnC were added to the fiber for another 20 min of incubation. Reconstituted strips were then washed in pCa 8 solution and subjected to force measurements. Efficiency of depletion and RLC reconstitution was tested by SDS-PAGE.

### The Ca^2+^ dependence of force development

Small porcine heart ventricular muscle strips of approximately 1.4 mm in length and 100 μm in diameter were attached by tweezer clips to a force transducer (Muthu et al., 2014). The strips were placed in a 1 ml cuvette and freshly skinned in 1% Triton X-100 dissolved in pCa 8 buffer (as mentioned above) for 30 min. They were rinsed 3 times × 5 min in pCa 8 buffer and their length adjusted to remove the slack. This procedure resulted in sarcomere length of ~2.1 μm as judged by the first order optical diffraction pattern as described in Wang et al. (2013a), Muthu et al. (2014). Then the strips were tested for maximal steady state force development in pCa 4 solution (composition is the same as pCa 8 buffer except the [Ca^2+^] = 10^−4^ M). Maximal tension readings at pCa 4 were taken before and after the force-pCa curve, averaged and expressed in kN/m^2^. The cross sectional area of the muscle strip was assumed to be circular. After the initial steady state force was determined, muscle strips were relaxed in pCa 8 buffer and exposed to solutions of increasing Ca^2+^ concentrations from pCa 8 to pCa 4. The level of force was measured in each “pCa” solution. Data were analyzed using the Hill equation (Hill et al., 1980):

(3) $$\begin{array}{r} f=y_{0}+\left(a{\left(10^{-x}\right)}^{b}\right)∕\left({\left(10^{-c}\right)}^{b}+{\left(10^{-x}\right)}^{b}\right) \end{array}$$

where *b* = n_H_ is the Hill coefficient, *c* = [Ca^2+^]_50_ or pCa_50_, is the free Ca^2+^ concentration which produces 50% of the maximal force. The pCa_50_ represents the measure of Ca^2+^ sensitivity of force and the n_H_ is the measure of myofilament cooperativity.

### Secondary structure prediction of WT and IVS6-1 RLCs

The secondary structure prediction was conducted with I-TASSER (online server from Zhanglab, University of Michigan): http://zhanglab.ccmb.med.umich.edu/I-TASSER/ as described earlier (Huang et al., 2015; Yuan et al., 2015). The amino acid sequence of IVS6-1-RLC was compared against template proteins selected from the PDB library of similar structures. The full length protein was assembled from the excised fragments and simulated into the lowest energy model using specific algorithms. The confidence of each predicted model structure was presented as C-score, ranging from −5 to 2. The quality of prediction was proportional to the value of C-score (Zhang, 2008; Roy et al., 2010, 2012). The predicted structures were then modeled using PyMOL molecular visualization system (Huang et al., 2015; Yuan et al., 2015).

### Statistical analysis

All values are shown as means ±SD (standard deviation) for n (number of independent experiments) ≤ 5 or ±SEM (standard error of the mean) for *n* ≥ 6. Statistically significant differences between two groups (WT and IVS6-1) were determined using an unpaired Student's *t*-test (Sigma Plot 11; Systat Software, San Jose, CA), with significance defined as *P* < 0.05.

## Results

### Molecular effects of IVS6-1RLC mutation

The IVS6-1 mutation originates from a frameshift within the *MYL2* gene and results in a replacement of the last 32 amino acids by 19 different amino acids, severely altering the C-terminus of the human cardiac RLC protein resulting in a shorter protein sequence (153 aa for IVS6-1 vs. 166 aa for WT) (Figure 1A). The purity of the recombinant RLC WT and IVS6-1 proteins was tested by Western blotting with antibodies against the C-terminus of RLC (CT-1) and its N-terminus (NT-1) (Figure 1B), both produced in this laboratory (Szczesna-Cordary et al., 2005; Wang et al., 2006). Due to the amino acid changes and the C-terminal truncation of IVS6-1, the mutant lost its C-terminal epitope and could only be detected with NT-1 (Figure 1B).

**Figure 2:**
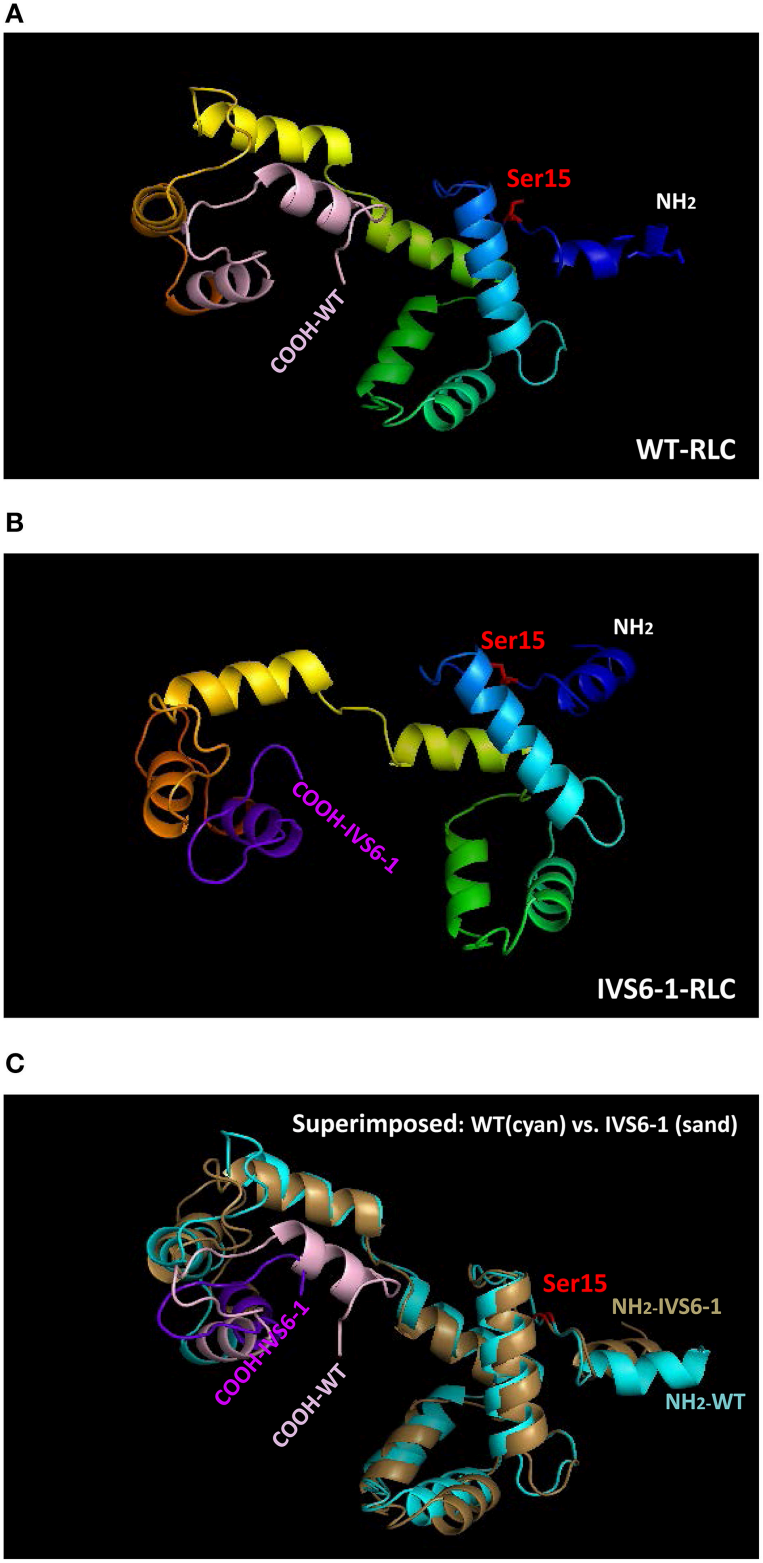
**Structure of human ventricular RLC-WT (A), RLC-IVS6-1 (B) predicted using I-TASSER, and the superimposed structures of RLC-WT (cyan) and RLC-IVS6-1 (sand) (C)**. The C-terminus of RLC-WT (light pink) is truncated and last 32 amino acids are replaced by 19 different amino acids (purple) in IVS6-1. The phosphorylation site of RLC at Ser15 is shown in red.

**Figure 3:**
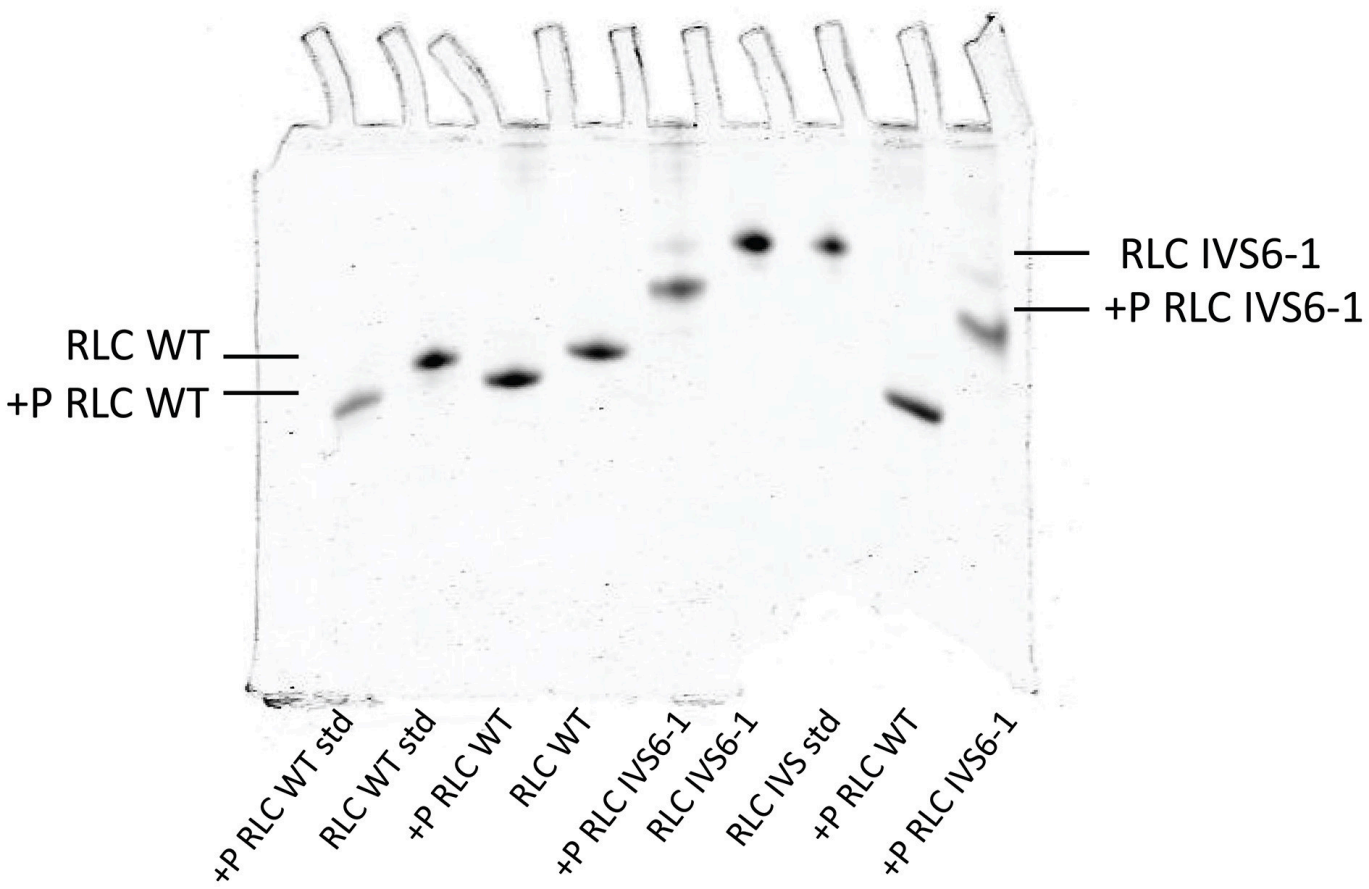
**Phosphorylation of IVS6-1 and WT RLCs with myosin light chain kinase (MLCK)**. Representative 8% UREA (8M) PAGE of phosphorylated vs. non-phosphorylated IVS6-1 and WT RLC proteins. 3-5 experiments were performed and the estimated level of phosphorylation was 92.7 ± 1.3% for IVS6-1 compared to 100% for WT RLC. Note the slower migration of IVS6-1 vs. WT even though the truncation mutation decreases the molecular weight of IVS6-1 (MW~17220 Da) compared with WT (MW~18789 Da). This is because the resultant pI of the IVS6-1 protein (predicted pI~5.50) is higher than that of WT (predicted pI~4.89).

To examine the effect of C-terminal truncation mutation on the secondary structure of the RLC, the I-TASSER computing program was used and the RLC-like protein templates extracted from the Protein Data Bank, as previously described (Huang et al., 2015). Structures with high similarity to the structure of RLC were used: PDB ID 4i2yA (chain A, crystal structure of the genetically encoded calcium indicator Rgeco1), PDB ID 3jvtB [chain B, calcium-bound scallop myosin regulatory domain (lever arm) with reconstituted complete light chains], PDB IF 3j04B (chain B, EM structure of the heavy meromyosin subfragment of Chick smooth muscle myosin with regulatory light chain in phosphorylated state), 1prwA (chain A, crystal structure of bovine brain Ca^2+^ calmodulin in a compact form), PDB ID 4ik1A (chain A, high-resolution structure of Gcampj at pH 8.5), PDB ID 2mysA (chain A, myosin subfragment 1) and PDB ID 2w4ab (chain B, isometrically contracting insect asynchronous flight muscle). The resulting modeled structures of RLC WT and IVS6-1 structures (Model with lowest C-score in I-TASSER) are presented in Figures 2A,B. Figure 2C shows the superimposed structures of WT and IVS6-1. The results show that the majority of changes occur in the C-terminal region of the RLC, leaving the structure of the N-terminus and the region linking the two RLC lobes unchanged (Figure 2). In addition, I-TASSER modeling data suggested that the RLC phosphorylation site at Ser15 is not affected by structural rearrangements of the C-terminus of RLC (Figure 2). We then pursued the investigation of the ability of IVS6-1 to become phosphorylated *in vitro* with the Ca^2+^-CaM activated MLCK (Figure 3). The slower band migration of IVS6-1 (MW~17220 Da) vs. WT (MW~18789 Da) was observed, and this was because of increased pI of the mutant (pI~5.50) compared to WT (pI~4.89). Likewise, phosphorylated forms of both WT and IVS6-1 migrated faster than their non-phosphorylated counterparts (Szczesna et al., 2001; Figure 3). The results indicated that Ser15 of IVS6-1-RLC could be phosphorylated by Ca^2+^-CaM MLCK as easily as WT RLC. However, these *in vitro* solution data may not directly translate to the *in situ* measures, when IVS6-1 is incorporated into the myosin lever arm in the thick filaments.

### The effect of IVS6-1 on RLC-MHC interaction

**Figure 4:**
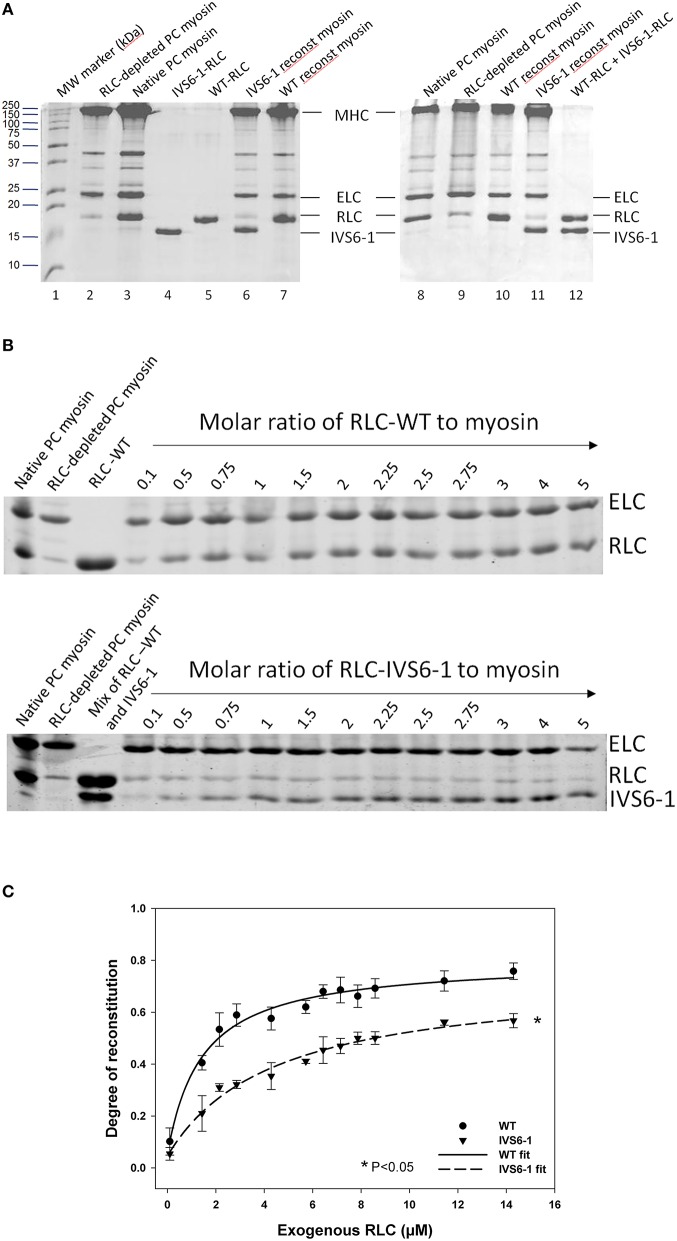
**The effect of IVS6-1 on the RLC-MHC interaction**. **(A)** Representative 15% SDS-PAGE images of native myosin, RLC-depleted myosin and WT or IVS6-1 reconstituted myosin. Lane 1, MW marker (kDa); lanes 2 and 9, RLC-depleted PC myosin; lanes 3 and 8, native PC myosin; lane 4, IVS6-1-RLC protein used in reconstitution; lane 5, WT-RLC used in reconstitution; lanes 6 and 11, IVS6-1 reconstituted PC myosin; lanes 7 and 10, WT reconstituted myosin; lane 12, mixture of WT and IVS6-1 proteins. **(B)** Titration experiments using RLC-depleted porcine cardiac myosin with increasing concentrations of WT or IVS6-1 RLCs. ELC (which remained intact during the depletion/reconstitution procedure) was used as the loading control. Numbers on the top indicate the molar ratio of RLC protein used for reconstitution to RLC-depleted PC myosin. **(C)** Binding isotherms of WT or IVS6-1 to RLC-depleted myosin. The data points were average of *n* = 4 experiments ± SD for WT, and *n* = 3 for IVS6-1. The data were fitted to the ligand- binding model Equation (1). Compared to WT, the maximal level of RLC reconstitution was significantly decreased in IVS6-1 and a significant decrease in the binding affinity to the MHC was observed for IVS6-1, ^*^P < 0.05.

To gain insight into the effect of IVS6-1 on the assembly of the RLC into the lever arm domain of MHC, we have studied the binding profiles of the WT and IVS6-1 proteins to the RLC-depleted porcine cardiac myosin. The CDTA/Triton-based treatment yielded >80% RLC-free myosin and the level of RLC remaining in RLC-depleted myosin was assessed by comparing the ratio of RLC/ELC bands in RLC-depleted to RLC/ELC of native myosin (Figure 4A). Titration experiments of RLC-depleted PC myosin (2.8 μM) incubated with increasing concentrations of human recombinant WT or IVS6-1 (from 0.1 μM to 14 μM) (Figure 4B) produced the binding isotherms (Figure 4C) and the Kd values of binding using Equation (1). We observed a significant reduction in Kd and the maximal level of reconstitution for IVS6-1 (Kd = 4.41 ± 0.79 (SD) μM and 57 ± 2%, *n* = 3) compared with WT (K_*d*_ = 1.42 ± 0.21 (SD) μM and 77 ± 2%, *n* = 4) (*P* < 0.05). These results suggested that the IVS6-1 truncation mutation was sufficient to impose severe conformational changes in the RLC structure that prevented the mutant to stoichiometrically bind to the MHC and structurally support the lever arm of myosin. These altered protein-protein interactions might be due to the mutant-induced changes in the tertiary structure of the RLC (Figure 2B) that ultimately trigger pathologic cardiac remodeling in the IVS6-1-mutated myocardium.

### Binding of RLC-IVS6-1 reconstituted myosin to pyrene labeled F-actin

**Figure 5:**
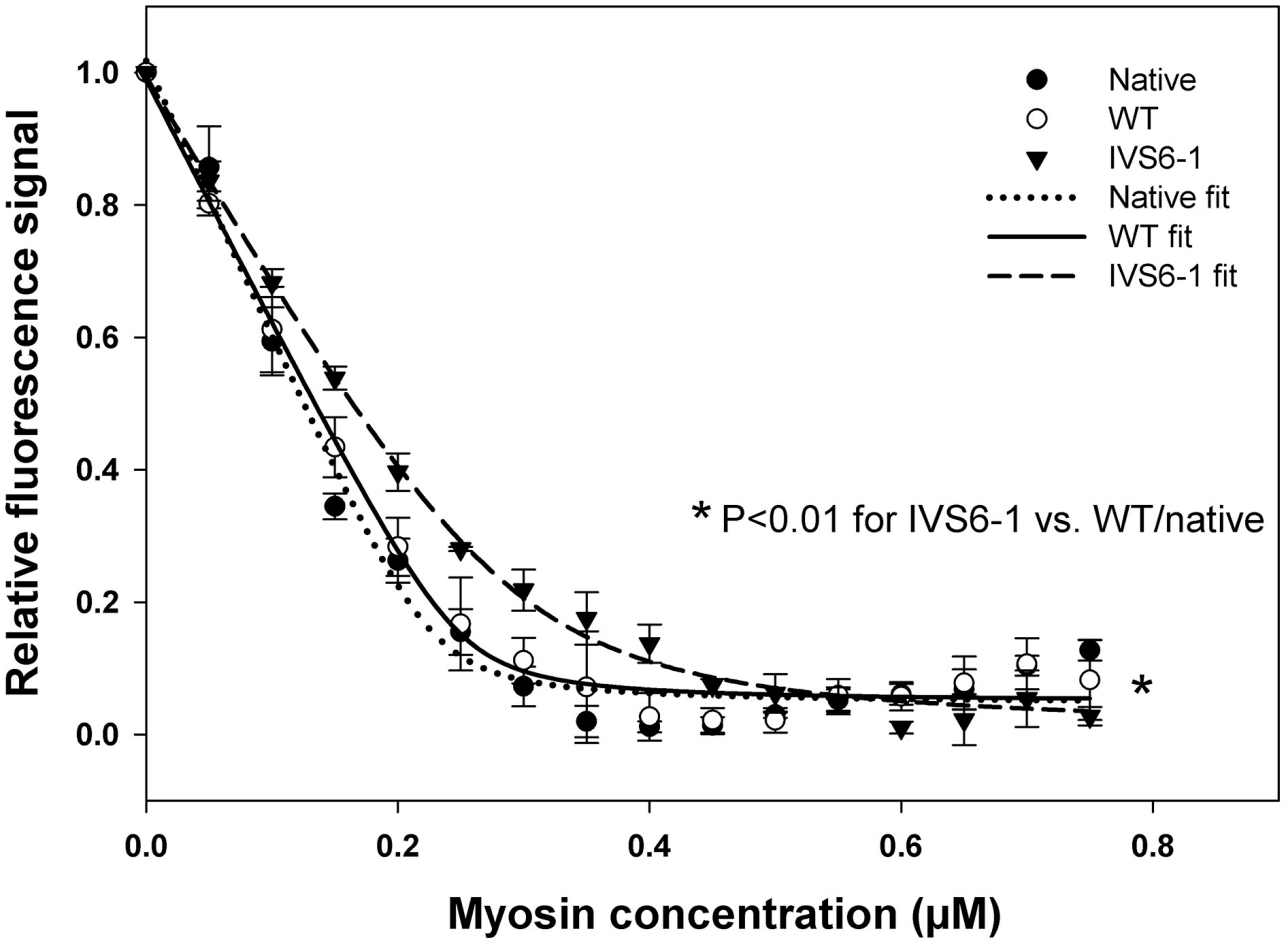
**Fluorescence steady-state binding of IVS6-1 mutant vs. WT -reconstituted porcine myosin to pyrene labeled F-actin under rigor conditions**. 0.5 μM pyrene-actin was titrated with increasing concentrations of native (black circles), WT (open circles) or IVS6-1 (black triangles) myosins. Note a significantly lower binding affinity of IVS6-1 reconstituted myosin to actin compared with WT or native myosins (^*^P < 0.01). Three to four experiments per group were performed.

Fluorescence steady-state binding of IVS6-1 mutant vs. WT reconstituted PC myosin to pyrene labeled F-actin was investigated under rigor conditions (Figure 5). Titration profiles of pyrene-actin with native PC myosin or WT-reconstituted myosin were not different while those of IVS6-1-reconstituted myosin showed impaired binding to actin. Titration data were fitted to Equation (2) to obtain the apparent dissociation constants (K_d_) and stoichiometry n of binding (Figure 5). The binding of PC myosin or WT/IVS6-1 reconstituted myosins to actin was strong (in nM range), but the mutant showed a lower binding affinity compared with WT or PC native myosin. The data for IVS6-1 showed: *K*_d_ = 16.7 ± 2.8 (SD) nM; stoichiometry *n* = 0.59±0.0.01 (*n* = 3), and for WT: *K*_d_ = 4.6 ± 1.3 (SD) nM; stoichiometry *n* = 0.51 ± 0.14 actin (*n* = 3). The binding affinity of native myosin, used as a control, for actin was *K*_d_ = 2.9 ± 0.9 (SD) nM with stoichiometry *n* = 0.47 ± 0.04 (*n* = 4). Therefore, IVS6-1 reduced the affinity of myosin for actin by ~4-fold compared to WT and by ~6-fold compared with native PC myosin (*P* < 0.01). There was no statistically significant difference in K_d_ between WT and native PC myosin (*P* = 0.103; Figure 5).

### Stopped flow measurements

**Figure 6:**
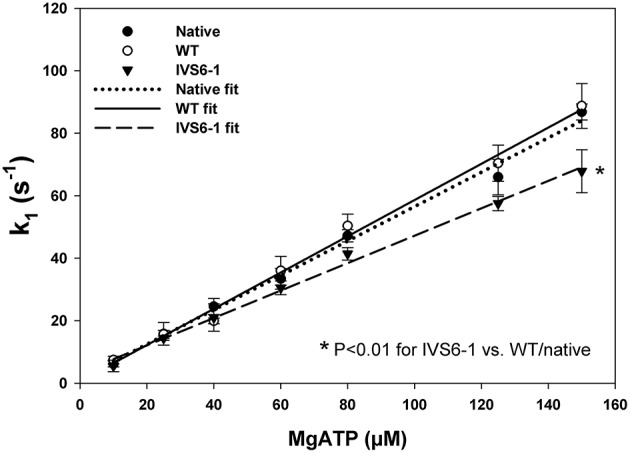
**The dissociation rate (k_1_)–[MgATP] dependence and the effective second-order MgATP binding rates (a = slope) for native porcine myosin and RLC-depleted porcine myosin reconstituted with WT, IVS6-1 complexed with F-actin**. The values of k_1_ ± SD for each MgATP concentration are presented in Table 1. Significantly altered binding rates were observed for IVS6-1 compared with WT (^*^P < 0.05). No statistically significant differences were observed between native myosin and WT-reconstituted myosin.

Fluorescence stopped-flow kinetic experiments were carried out on myosin reconstituted with RLC-WT or –IVS6-1 and pyrene labeled F-actin to further examine the effects of IVS6-1 on the interaction of myosin with actin. The time course of the recovery in the pyrene fluorescence was monitored as a function of Mg-ATP concentrations. The Mg-ATP-dependent transition of the strongly bound acto-myosin complex (M•A) to the weakly bound state (M•A•ATP) was measured by mixing actin-myosin complexes in a 1:1 vol/vol ratio with increasing concentrations of Mg-ATP (10–150 μM) in a stopped flow apparatus. An increase in the fluorescence intensity on the addition of MgATP was monitored as a function of time (not shown) as the myosin heads dissociated from pyrene-F-actin on the addition of MgATP. The observed actin-myosin dissociation rate constant (k_1_) for the MA to MAATP transition was derived from the averaged fluorescence traces and fitted with a single exponential dependence. The values of k_1_ ± SD for each MgATP concentration are presented in Table 1. The results revealed significant differences in k_1_ between IVS6-1- and WT-reconstituted myosins for 80, 125, and 150 μM Mg-ATP concentration, indicating slower dissociation rates in IVS6-1 compared with WT. A plot of the observed transition rates (k_1_) as a function of [MgATP] is presented in Figure 6, which showed linear-type of dependence with the slope “a” value corresponding to the effective second-order Mg-ATP binding rates. Significantly altered binding rates were observed for IVS6-1 compared with WT/native myosins (*n* = 3–5 experiments per group) with binding rates (in M^−1^s^−1^): 4.3 ± 0.01 × 10^5^ (IVS6-1) vs. 5.8 ± 0.02 × 10^5^ (WT) vs. 5.5 ± 0.02 × 10^5^ (native) (*P* < 0.01). No statistically significant differences were observed between native myosin and WT-reconstituted myosin.

**Table 1:** **Stopped-flow kinetics of MgATP induced actin-myosin dissociation**.

**Samples**	**MgATP concentration**						
	**10 μm**	**25 μm**	**40 μm**	**60 μm**	**80 μm**	**125 μm**	**150 μm**
Native	6.2 ± 0.9	15.8 ± 3.6	24.5 ± 2.6	33.4 ± 3.3	47.2 ± 1.9	65.9 ± 5.7	86.8 ± 2.6
WT	7.4 ± 1.3	15.6 ± 1.3	19.9 ± 0.9	36.0 ± 4.6	50.4 ± 3.7	70.4 ± 5.8	88.7 ± 7.1
IVS6-1	5.5 ± 1.8	14.4 ± 0.7	21.0 ± 4.4	30.5 ± 2.2	41.4 ± 1.9^a^	57.5 ± 2.3^a^	67.8 ± 6.8^a^

Pyrene-labeled F-actin was complexed with IVS6-1 or WT –reconstituted myosin. The dissociation rates k_1_ were in s^−1^, n = 5–10.

a P < 0.05 compared with WT.

### Actin activated myosin ATPase activity

**Figure 7:**
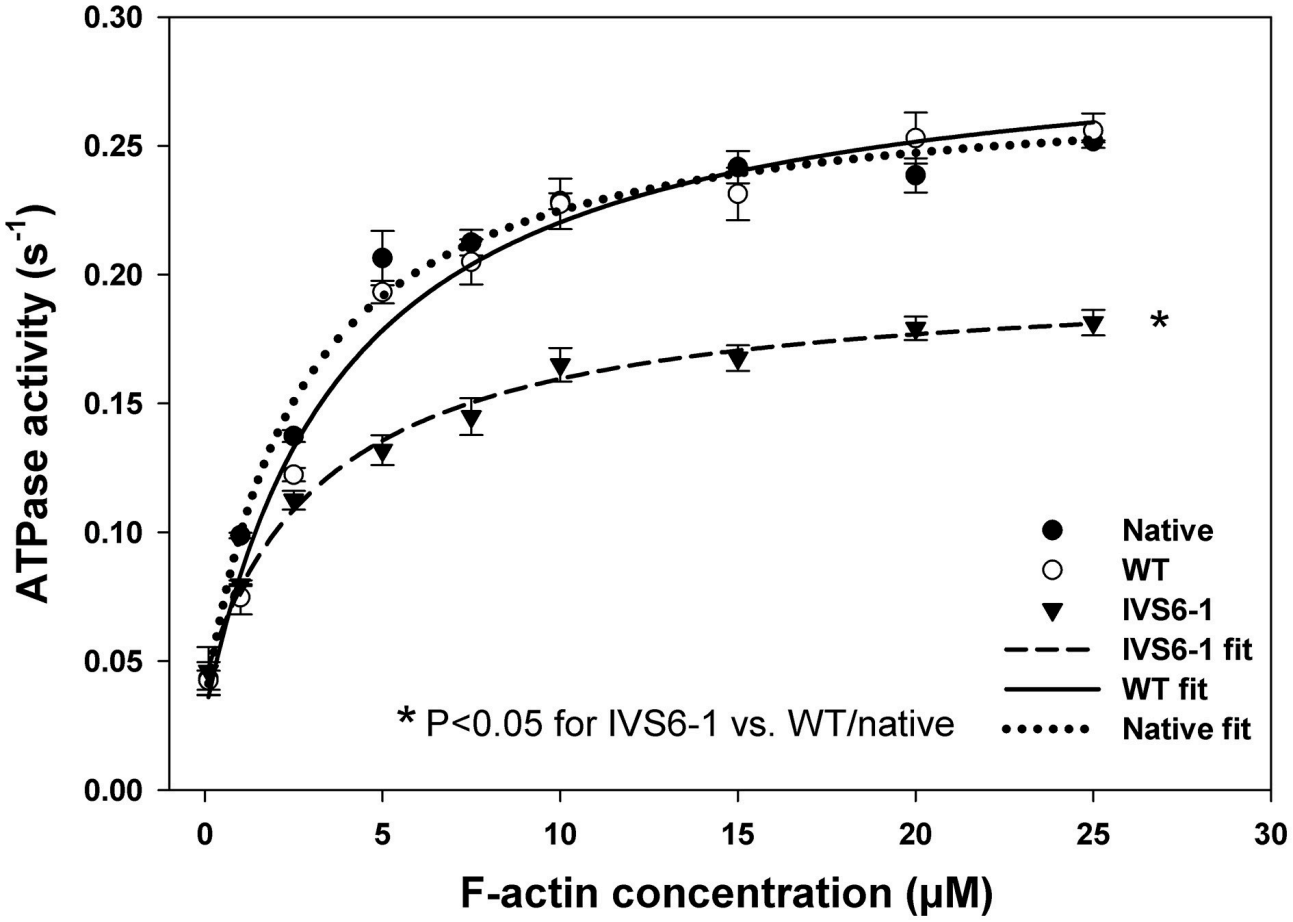
**The effect of splice site RLC mutation on the actin-activated myosin ATPase activity**. Note that IVS6-1 (black triangles) resulted in significantly lower Vmax compared with native porcine myosin (black circles) and WT reconstituted myosin (open circles). Porcine cardiac myosin followed the same procedures that were used for experimental myosins and served as a control. The data are the average of *n* = 3–4 experiments for each group of myosins ±SD. The “^*^” symbol denotes significant changes with *P* < 0.05.

To identify the IVS6-1-induced changes in actin-myosin interaction, actin-activated myosin ATPase activity assays were carried out as a function of actin concentration (in μM) using porcine myosin reconstituted with WT or IVS6-1 RLCs. The acto-myosin ATPase profiles of IVS6-1 mutant, WT and native myosin control are shown in Figure 7. The data are the average of *n* = 3–4 individual experiments ±SD and analyzed using the Michaelis–Menten equation yielding the V_max_ and K_m_ parameters (Trybus, 2000). The V_*max*_ represents the rate constant of the detachment step and the transition from the weakly (A·M·ATP↔A·M·ADP·Pi) to strongly (A·M·ADP↔A·M) bound cross-bridges (Kazmierczak et al., 2012). The IVS6-1 demonstrated significantly decreased V_max_ = 0.15 ± 0.01 s^−1^ (*n* = 3) compared with WT: V_max_ = 0.25 ± 0.01 s^−1^ (*n* = 3) and native myosin: V_max_ = 0.24 ± 0.01 (*n* = 4) s^−1^ (Figure 7, *P* < 0.05). No statistically significant differences were observed between native PC and WT-reconstituted myosins. The results suggested that IVS6-1 mutation may slow down the ATPase cycle or may decrease the number of cycling cross-bridges during muscle contraction. The K_m_ (in μM) ±SD values were 1.93 ± 0.06, 3.67 ± 0.23, and 3.33 ± 0.57 for native PC, WT-, and IVS6-1 reconstituted myosins. No statistical significance was noted between WT and IVS6-1 (*P* > 0.05).

### Steady-state force and force-pCa relationship in WT- and IVS6-1- reconstituted porcine papillary muscle strip

**Figure 8:**
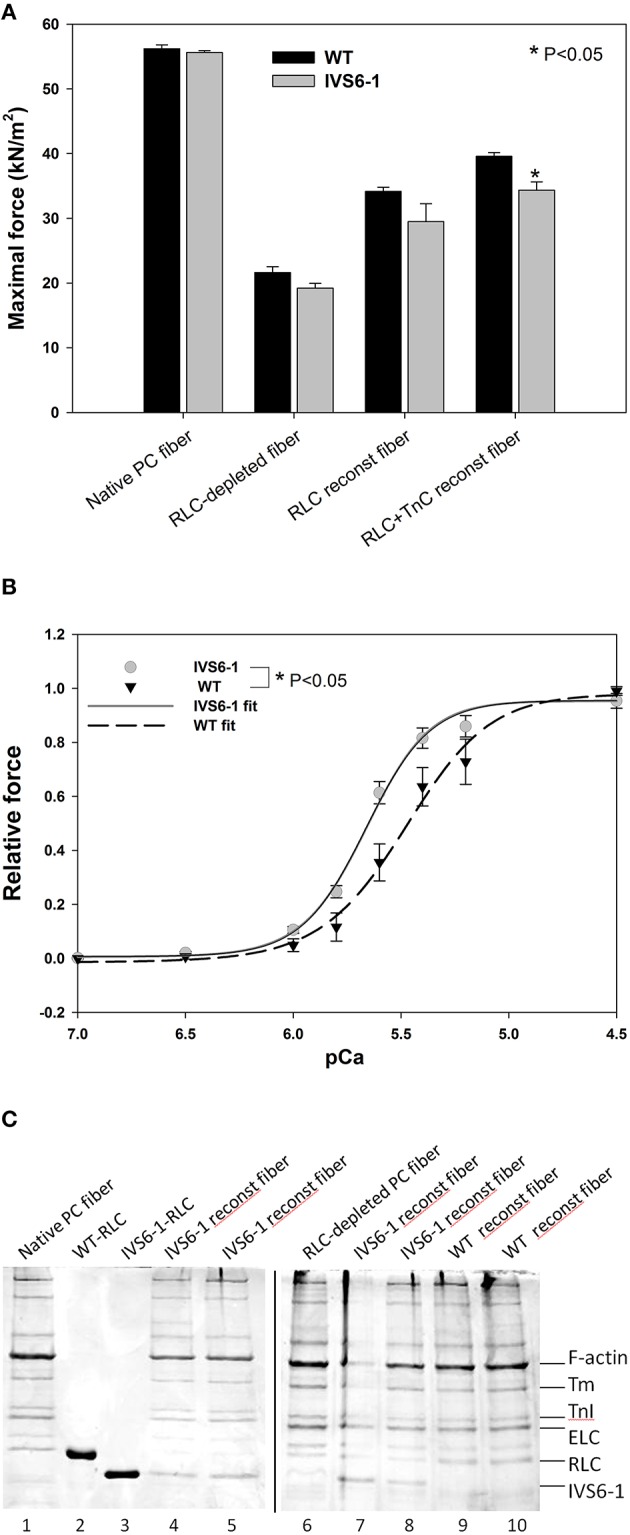
**(A)** Maximal force measured in pCa 4 after RLC and TnC reconstitution in porcine papillary muscle fibers. Note that IVS6-1 imposed a significant reduction in maximal level of tension in IVS6-1 reconstituted fibers (*n* = 6) compared with WT reconstituted fibers (*n* = 8) (^*^P < 0.05). The average diameter of muscle strips (in μm) was 89 ± 5 for IVS6-1 and 101 ± 5 for WT. **(B)** IVS6-1- induced increase in Ca^2+^ sensitivity of force. The number of experiments as in A. There was a significant difference between pCa_50_ of IVS6-1 vs. WT reconstituted fibers (^*^P < 0.05). **(C)** Representative 15% SDS-PAGE of CDTA depleted and RLC/TnC reconstituted porcine papillary muscle strips. Lane 1, native PC fiber; lane 2, recombinant human cardiac WT-RLC protein used for fiber reconstitution; lane 3, recombinant human cardiac IVS6-1-RLC protein used for fiber reconstitution; lanes 4, 5, 7, and 8, IVS6-1 reconstituted fibers; lane 6, RLC-depleted fiber; lanes 9 and 10, WT-reconstituted fibers.

To further assess the effects of IVS6-1 on cardiac muscle contraction, the RLC WT or IVS6-1 -reconstituted skinned porcine papillary muscle strips were subjected to force-pCa measurements. A significant decrease in maximal isometric force was observed in fibers reconstituted with IVS6-1 compared with WT (Figure 8A). The values of force per cross-sectional area of muscle (in kN/m^2^ ± SEM) were: IVS6-1, 27 ± 1.0 (*n* = 6) vs. WT, 35 ± 2.0 (*n* = 8). The average diameter of muscle strips (in μm) was 89 ± 5 for IVS6-1 and 101 ± 5 for WT. The data of force-pCa measurements were plotted and fitted using the Hill equation (Equation 3). There was a statistically significant increase in pCa_50_ of the force-pCa dependence: pCa_50_ = 5.66 ± 0.01 observed for IVS6-1 compared with 5.48 ± 0.01 for WT (Figure 8B, *P* < 0.05). The IVS6-1 mutation also affected the Hill coefficient, and 2.90 ± 0.15 was observed for IVS6-1 and 2.20 ± 0.14 for WT (Figure 8B). The efficiency of RLC-depletion and reconstitution with RLC/IVS6-1 proteins was tested by SDS-PAGE and is shown in Figure 8C. On average, more than 80% of RLC-depletion, and near 100% fiber reconstitution was observed for both WT and IVS6-1 proteins. The results from functional studies indicated that IVS6-1 was able to bind to the lever arm domain of myosin cross-bridge and impose significant alterations in the force-pCa dependence and in the ability of myosin to develop maximal isometric force.

## Discussion

Myosin regulatory and essential light chains bind to the myosin heavy chain at the lever arm domain (Rayment et al., 1993b; Geeves, 2002) and structurally support this region of the myosin head contributing to its stiffness and cross-bridge compliance (Muthu et al., 2011; Wang et al., 2013a,b). They also actively participate in the ATP-powered myosin cross-bridge cycle and muscle contraction (Rayment et al., 1993a; Geeves and Holmes, 2005). The cardiac myosin RLC belongs to the superfamily of the EF-hand Ca^2+^-binding proteins and its N-terminal tail contains one Ca^2+^-Mg^2+^ binding site (Lowey and Risby, 1971; Alexis and Gratzer, 1978). It has been postulated that during cardiac muscle contraction, this RLC site may work as a delayed Ca^2+^ buffer helping SERCA2a pump Ca^2+^ back to the SR (sarcoplasmic reticulum) during diastole (Wang et al., 2006; Szczesna-Cordary et al., 2007). The N-terminus of cardiac RLC also contains the Ca^2+^/CaM-MLCK dependent phosphorylation site at Ser15, which can become phosphorylated when Ca^2+^ is released from the SR and activates the myosin light chain kinase (Kamm and Stull, 2001). Our previous research showed that the properties of both of these two functional sites (Ca^2+^-binding site and phosphorylation site) of the RLC can be significantly altered in the presence of cardiomyopathy-associated mutations in *MYL2* (Szczesna et al., 2001; Szczesna-Cordary et al., 2004).

In this report we describe a novel *MYL2* mutation, which was recently identified to be responsible for a long known cardioskeletal myopathy observed in Dutch and Italian families (Barth et al., 1998; Weterman et al., 2013) The genetic cause for this hereditary disorder resulting in skeletal and cardiac muscle myopathy was found to be due to mutations in the gene encoding for the ventricular and slow twitch skeletal isoforms of the RLC, *MYL2* (Weterman et al., 2013). The most severe of all was the splice site mutation located in the intron 6 of *MYL2* (IVS6-1) that ultimately resulted in the C-terminal truncation of the RLC protein changing its amino acid sequence at the C-terminal tail of RLC (Weterman et al., 2013). The homozygous appearance of IVS6-1 led to the early death of infants (4–6 months of age) due to dilated (DCM), hypertrophic (HCM) or non-compaction cardiomyopathy, while no obvious phenotype was noted in family members heterozygous for IVS6-1 (Barth et al., 1998; Weterman et al., 2013). The homozygous patients demonstrated dual cardiac and skeletal muscle myopathy with morphological features of muscle type I hypotrophy and the skeletal and cardiac myofibril disorganization.

Here, we have examined the molecular and functional consequences of IVS6-1 *in vitro* using recombinant IVS6-1 and wild-type RLC proteins that could be reconstituted into RLC-depleted porcine cardiac muscle preparations (PC myosin, acto-myosin complex and skinned papillary muscle strips). As the IVS6-1 mutation arose from a frameshift in the MYL2 gene, the resultant protein demonstrated a replacement of the last 32 amino acids by 19 different amino acids. The molecular analysis of the mutation-induced conformational changes in the RLC molecule using I-TASSER computation (Huang et al., 2015; Yuan et al., 2015) clearly showed the C-terminal RLC molecular rearrangements due to IVS6-1. The observed differences between WT RLC and the mutant with the latter being 13-amino acid shorter were observed in the newly formed C-terminus of IVS6-1.

Interestingly, IVS6-1 did not eliminate the ability for MLCK-induced phosphorylation of the RLC *in vitro*, which was not surprising given that the accessibility of Ser15 was not observed to be obstructed by the C-terminal RLC truncation in IVS6-1. *In vivo*, the RLC phosphorylation was shown to be a significant modulatory mechanism of myosin activation and muscle contraction, and a severely decreased level of RLC phosphorylation was observed in the hearts of HCM and/or heart failure patients (Van Der Velden et al., 2003a,b,c) and in the animal models of HCM (Abraham et al., 2009; Muthu et al., 2010, 2012; Yuan et al., 2015). Future studies will have to be executed to examine the effect of IVS6-1 on myosin RLC phosphorylation *in vivo*.

Since the C-terminal region of the RLC is involved in its interaction with the myosin heavy chain (Rayment et al., 1993b), we proceeded to examine the effect of IVS6-1 truncation mutation on the incorporation of the mutant RLC into the myosin lever arm domain. The data revealed that the mutation was sufficient to disrupt the RLC-MHC interaction and reduce the K_d_ of binding. This altered RLC-MHC interaction in the mutant was most likely responsible for the significant changes that we observed in the interaction of the mutant-reconstituted myosin and actin. While no differences in the binding profile to pyrene-labeled F-actin between the native or WT-reconstituted PC myosin were observed, the affinity of IVS6-1 -reconstituted myosin for pyrene-actin was ~4-fold lower compared with WT. Therefore, the truncation mutation of the RLC and the changes in the amino acid sequence of its new C-terminus resulted in a significantly reduced affinity of IVS6-1-mutant myosin to F-actin. Likewise, the stopped-flow kinetics of the myosin–actin interaction were significantly reduced with decreased slope of the kobs-[MgATP] relationship for IVS6-1-reconstituted myosin compared with WT. These results suggest that IVS6-1 mutation may not only lower the affinity of the myosin-actin binding, but also reduces the kinetics of the ATP-induced dissociation of IVS6-1 myosin from actin. Results from actin-activated myosin ATPase activity assays and significantly decreased Vmax (by 1.7-fold compared with WT) are in agreement with stopped-flow data. These *in vitro* results may explain what has been observed in skinned muscle fiber strips where the calcium sensitivity of tension was significantly increased and the maximal level of tension was significantly reduced in IVS6-1-recnstituted fibers compared with WT. Interestingly, the effects on contractile force and myofilament calcium sensitivity observed in this study were similar to previously reported effects of other HCM-associated mutations in myosin RLC, investigated in transgenic RLC mice (Abraham et al., 2009; Kerrick et al., 2009; Yuan et al., 2015). Our collective results suggest that the IVS6-1 mutation may lead to cardiac dysfunction by disrupting the RLC-MHC and acto-myosin interactions (steady-state and kinetics) ultimately leading to compromised ability of the mutant myosin to develop contractile force and sensitizing myofilaments to calcium, effects that are hallmarks of HCM disease.

## Limitations, concluding remarks and future directions

An important issue which was not addressed experimentally here is the effect of IVS6-1 mutation in heterozygous state with 50:50 ratio of WT and IVS6-1 proteins. Heterozygous patients for IVS6-1± have no cardiomyopathy or slow-twitch skeletal myopathy symptoms; however, no data on protein expression were presented in the heterozygous parents of IVS6-1^+∕+^ toddlers (Weterman et al., 2013). Higher than 80% IVS6-1 reconstitution, achieved in porcine cardiac muscle preparations, most likely resembles the homozygous state. Our results suggest that when placed *in vivo* IVS6-1^+∕+^ may lead to diastolic and systolic dysfunction by delaying muscle relaxation, increasing calcium sensitivity of contraction and reducing maximal force generation. These speculations are supported by measurements of the acto-myosin kinetics and the observation of slower myosin cross-bridge turnover rates and slower second-order MgATP binding rates in IVS6-1 vs. WT reconstituted PC cardiac myosin. Our previous studies of the D166V RLC mutation, located at the last amino acid residue of the human cardiac RLC had shown similar effects on force generation in skinned papillary muscle fibers from the hearts of transgenic mice, i.e., significantly increased the Ca^2+^-sensitivity of contraction, diminished maximal tension and delayed muscle relaxation (Kerrick et al., 2009). These observations in skinned papillary muscle fibers from D166V mice were further confirmed by echocardiography and invasive hemodynamics showing systolic and diastolic dysfunction in D166 mice (Yuan et al., 2015). Future studies on IVS6-1 animal models are necessary to associate this truncation mutation in the RLC with cardiac dysfunction causing the early death of IVS6-1^+∕+^ infants.

## Author contributions

ZZ, DSC conceived and designed research, analyzed and interpreted data, prepared figures and drafted the manuscript; ZZ, WH, JL conducted experiments; WH cloned IVS6-1-*MYL2* and performed I-TASSER computation. DSC prepared the manuscript for publication and approved the revised version of the manuscript.

### Conflict of interest statement

The authors declare that the research was conducted in the absence of any commercial or financial relationships that could be construed as a potential conflict of interest.

## Abbreviations

CD: Circular Dichroism
DCM: dilated cardiomyopathy
ELC: essential light chain of myosin
HCM: hypertrophic cardiomyopathy
IVS6-1: splice site intronic mutation in MYL2
MHC: myosin heavy chain
MYL2: gene encoding for human ventricular RLC
RLC: regulatory light chain of myosin
Tm: tropomyosin
Tn: troponin
WT: wild-type.

## References

[B1] Abraham T. P. Jones M. Kazmierczak K. Liang H.-Y. Pinheiro A. C. Wagg C. S.. (2009). Diastolic dysfunction in familial hypertrophic cardiomyopathy transgenic model mice. Cardiovasc. Res. 82, 84–92. 10.1093/cvr/cvp016 19150977 PMC2721639

[B2] Alexis M. N. Gratzer W. B. (1978). Interaction of skeletal myosin light chains with calcium ions. Biochemistry 17, 2319–2325. 10.1021/bi00605a010 678511

[B3] Andersen P. S. Havndrup O. Bundgaard H. Moolman-Smook J. C. Larsen L. A. Mogensen J.. (2001). Myosin light chain mutations in familial hypertrophic cardiomyopathy: phenotypic presentation and frequency in Danish and South African populations. J. Med. Genet. 38:e43. 10.1136/jmg.38.12.e43 11748309 PMC1734772

[B4] Andersen P. S. Havndrup O. Hougs L. Sørensen K. M. Jensen M. Larsen L. A.. (2009). Diagnostic yield, interpretation, and clinical utility of mutation screening of sarcomere encoding genes in Danish hypertrophic cardiomyopathy patients and relatives. Hum. Mutat. 30, 363–370. 10.1002/humu.20862 19035361

[B5] Barth P. G. Wanders R. J. Ruitenbeek W. Roe C. Scholte H. R. Van Der Harten H.. (1998). Infantile fibre type disproportion, myofibrillar lysis and cardiomyopathy: a disorder in three unrelated Dutch families. Neuromuscul. Disord. 8, 296–304. 10.1016/S0960-8966(98)00028-5 9673982

[B6] Claes G. R. Van Tienen F. H. Lindsey P. Krapels I. P. Helderman-Van Den Enden A. T. Hoos M. B.. (2015). Hypertrophic remodelling in cardiac regulatory myosin light chain *(MYL2)* founder mutation carriers. Eur. Heart J. 10.1093/eurheartj/ehv522. [Epub ahead of print]. 26497160

[B7] Cooper A. Walker S. B. Pollard T. D. (1983). Pyrene actin: documentation of the validity of a sensitive assay for actin polymerization. J. Muscle Res. Cell Mot. 4, 253–262. 10.1007/BF00712034 6863518

[B8] Farman G. P. Muthu P. Kazmierczak K. Szczesna-Cordary D. Moore J. R. (2014). Impact of familial hypertrophic cardiomyopathy-linked mutations in the NH_2_-terminus of the RLC on β-myosin cross-bridge mechanics. J. Appl. Physiol. 117, 1471–1477. 10.1152/japplphysiol.00798.2014 25324513 PMC4269682

[B9] Fiske C. H. Subbarow Y. (1925). The colorimetric determination of phosphorus. J. Biol. Chem. 66, 375–400.

[B10] Flavigny J. Richard P. Isnard R. Carrier L. Charron P. Bonne G.. (1998). Identification of two novel mutations in the ventricular regulatory myosin light chain gene (MYL2) associated with familial and classical forms of hypertrophic cardiomyopathy. J. Mol. Med. 76, 208–214. 10.1007/s001090050210 9535554

[B11] Garcia-Pavia P. Vázquez M. E. Segovia J. Salas C. Avellana P. Gómez-Bueno M.. (2011). Genetic basis of end-stage hypertrophic cardiomyopathy. Eur. J. Heart Fail. 13, 1193–1201. 10.1093/eurjhf/hfr110 21896538

[B12] Geeves M. A. (2002). Molecular motors: stretching the lever-arm theory. Nature 415, 129–131. 10.1038/415129a 11805818

[B13] Geeves M. A. Holmes K. C. (2005). The molecular mechanism of muscle contraction. Adv. Protein Chem. 71, 161–193. 10.1016/S0065-3233(04)71005-0 16230112

[B14] Greenberg M. J. Kazmierczak K. Szczesna-Cordary D. Moore J. R. (2010). Cardiomyopathy-linked myosin regulatory light chain mutations disrupt myosin strain-dependent biochemistry. Proc. Natl. Acad. Sci. U.S.A. 107, 17403–17408. 10.1073/pnas.1009619107 20855589 PMC2951453

[B15] Greenberg M. J. Mealy T. R. Watt J. D. Jones M. Szczesna-Cordary D. Moore J. R. (2009). The molecular effects of skeletal muscle myosin regulatory light chain phosphorylation. Am. J. Physiol. Regul. Integr. Comp. Physiol. 297, R265–R274. 10.1152/ajpregu.00171.2009 19458282 PMC2724231

[B16] Hill T. L. Einsenberg E. Greene L. E. (1980). Theoretical model for the cooperative equilibrium binding of myosin subfragment-1 to the actin-troponin-tropomyosin complex. Proc. Natl. Acad. Sci. U.S.A. 77, 3186–3190. 10.1073/pnas.77.6.3186 10627230 PMC349579

[B17] Holmes K. C. Geeves M. A. (2000). The structural basis of muscle contraction. Philos. Trans. R. Soc. B Biol. Sci. 355, 419–431. 10836495 10.1098/rstb.2000.0583 PMC1692754

[B18] Huang W. Liang J. Yuan C. C. Kazmierczak K. Zhou Z. Morales A.. (2015). Novel familial dilated cardiomyopathy mutation in MYL2 affects the structure and function of myosin regulatory light chain. FEBS J. 282, 2379–2393. 10.1111/febs.13286 25825243 PMC4472530

[B19] Kabaeva Z. T. Perrot A. Wolter B. Dietz R. Cardim N. Correia J. M.. (2002). Systematic analysis of the regulatory and essential myosin light chain genes: genetic variants and mutations in hypertrophic cardiomyopathy. Eur. J. Hum. Genet. 10, 741–748. 10.1038/sj.ejhg.5200872 12404107

[B20] Kamm K. E. Stull J. T. (2001). Dedicated myosin light chain kinases with diverse cellular functions. J. Biol. Chem. 276, 4527–4530. 10.1074/jbc.R000028200 11096123

[B21] Karabina A. Kazmierczak K. Szczesna-Cordary D. Moore J. R. (2015). Myosin regulatory light chain phosphorylation enhances cardiac beta-myosin *in vitro* motility under load. Arch. Biochem. Biophys. 580, 14–21. 10.1016/j.abb.2015.06.014 26116789 PMC4790447

[B22] Kazmierczak K. Muthu P. Huang W. Jones M. Wang Y. Szczesna-Cordary D. (2012). Myosin regulatory light chain mutation found in hypertrophic cardiomyopathy patients increases isometric force production in transgenic mice. Biochem. J. 442, 95–103. 10.1042/BJ20111145 22091967 PMC6589164

[B23] Kazmierczak K. Xu Y. Jones M. Guzman G. Hernandez O. M. Kerrick W. G. L.. (2009). The role of the N-Terminus of the myosin essential light chain in cardiac muscle contraction. J. Mol. Biol. 387, 706–725. 10.1016/j.jmb.2009.02.006 19361417 PMC3068778

[B24] Kerrick W. G. L. Kazmierczak K. Xu Y. Wang Y. Szczesna-Cordary D. (2009). Malignant familial hypertrophic cardiomyopathy D166V mutation in the ventricular myosin regulatory light chain causes profound effects in skinned and intact papillary muscle fibers from transgenic mice. FASEB J. 23, 855–865. 10.1096/fj.08-118182 18987303 PMC2653985

[B25] Lowey S. Risby D. (1971). Light chains from fast and slow muscle myosins. Nature 234, 81–85. 10.1038/234081a0 4942892

[B26] Muthu P. Kazmierczak K. Jones M. Szczesna-Cordary D. (2012). The effect of myosin RLC phosphorylation in normal and cardiomyopathic mouse hearts. J. Cell. Mol. Med. 16, 911–919. 10.1111/j.1582-4934.2011.01371.x 21696541 PMC3193868

[B27] Muthu P. Liang J. Schmidt W. Moore J. R. Szczesna-Cordary D. (2014). *In vitro* rescue study of a malignant familial hypertrophic cardiomyopathy phenotype by pseudo-phosphorylation of myosin regulatory light chain. Arch. Biochem. Biophys. 552-553, 29–39. 10.1016/j.abb.2013.12.011 24374283 PMC4043912

[B28] Muthu P. Mettikolla P. Calander N. Luchowski R. Gryczynski I. Gryczynski Z.. (2010). Single molecule kinetics in the familial hypertrophic cardiomyopathy D166V mutant mouse heart. J. Mol. Cell. Cardiol. 48, 989–998. 10.1016/j.yjmcc.2009.11.004 19914255 PMC2854267

[B29] Muthu P. Wang L. Yuan C. C. Kazmierczak K. Huang W. Hernandez O. M.. (2011). Structural and functional aspects of the myosin essential light chain in cardiac muscle contraction. FASEB J. 25, 4394–4405. 10.1096/fj.11-191973 21885653 PMC3236635

[B30] Olivotto I. Girolami F. Ackerman M. J. Nistri S. Bos J. M. Zachara E.. (2008). Myofilament protein gene mutation screening and outcome of patients with hypertrophic cardiomyopathy. Mayo Clin. Proc. 83, 630–638. 10.1016/S0025-6196(11)60890-2 18533079

[B31] Pant K. Watt J. Greenberg M. Jones M. Szczesna-Cordary D. Moore J. R. (2009). Removal of the cardiac myosin regulatory light chain increases isometric force production. FASEB J. 23, 3571–3580. 10.1096/fj.08-126672 19470801 PMC2747675

[B32] Pardee J. D. Spudich J. A. (1982). Purification of muscle actin. Methods Enzymol 85 Pt B, 164–181. 10.1016/0076-6879(82)85020-9 7121269

[B33] Poetter K. Jiang H. Hassanzadeh S. Master S. R. Chang A. Dalakas M. C.. (1996). Mutations in either the essential or regulatory light chains of myosin are associated with a rare myopathy in human heart and skeletal muscle. Nat. Genet. 13, 63–69. 10.1038/ng0596-63 8673105

[B34] Rayment I. Holden H. M. Whittaker M. Yohn C. B. Lorenz M. Holmes K. C.. (1993a). Structure of the actin-myosin complex and its implications for muscle contraction. Science 261, 58–65. 10.1126/science.8316858 8316858

[B35] Rayment I. Rypniewski W. R. Schmidt-Bäse K. Smith R. Tomchick D. R. Benning M. M.. (1993b). Three-dimensional structure of myosin subfragment-1: a molecular motor. Science 261, 50–58. 10.1126/science.8316857 8316857

[B36] Richard P. Charron P. Carrier L. Ledeuil C. Cheav T. Pichereau C.. (2003). Hypertrophic cardiomyopathy: distribution of disease genes, spectrum of mutations, and implications for a molecular diagnosis strategy. Circulation 107, 2227–2232. 10.1161/01.CIR.0000066323.15244.54 12707239

[B37] Roy A. Kucukural A. Zhang Y. (2010). I-TASSER: a unified platform for automated protein structure and function prediction. Nat. Protoc. 5, 725–738. 10.1038/nprot.2010.5 20360767 PMC2849174

[B38] Roy A. Yang J. Zhang Y. (2012). COFACTOR: an accurate comparative algorithm for structure-based protein function annotation. Nucleic Acids Res. 40, W471–W477. 10.1093/nar/gks372 22570420 PMC3394312

[B39] Szczesna D. Ghosh D. Li Q. Gomes A. V. Guzman G. Arana C.. (2001). Familial hypertrophic cardiomyopathy mutations in the regulatory light chains of myosin affect their structure, Ca^2+^ binding, and phosphorylation. J. Biol. Chem. 276, 7086–7092. 10.1074/jbc.M009823200 11102452

[B40] Szczesna-Cordary D. Guzman G. Ng S. S. Zhao J. (2004). Familial hypertrophic cardiomyopathy-linked alterations in Ca^2+^ binding of human cardiac myosin regulatory light chain affect cardiac muscle contraction. J. Biol. Chem. 279, 3535–3542. 10.1074/jbc.M307092200 14594949

[B41] Szczesna-Cordary D. Guzman G. Zhao J. Hernandez O. Wei J. Diaz-Perez Z. (2005). The E22K mutation of myosin RLC that causes familial hypertrophic cardiomyopathy increases calcium sensitivity of force and ATPase in transgenic mice. J. Cell Sci. 118, 3675–3683. 10.1242/jcs.02492 16076902

[B42] Szczesna-Cordary D. Jones M. Moore J. R. Watt J. Kerrick W. G. L. Xu Y.. (2007). Myosin regulatory light chain E22K mutation results in decreased cardiac intracellular calcium and force transients. FASEB J. 21, 3974–3985. 10.1096/fj.07-8630com 17606808

[B43] Trybus K. M. (2000). Biochemical studies of myosin. Methods 22, 327–335. 10.1006/meth.2000.1085 11133239

[B44] Van Der Velden J. Papp Z. Boontje N. M. Zaremba R. De Jong J. W. Janssen P. M.. (2003a). Myosin light chain composition in non-failing donor and end-stage failing human ventricular myocardium. Adv. Exp. Med. Biol. 538, 3–15. 10.1007/978-1-4419-9029-7_1 15098650

[B45] Van Der Velden J. Papp Z. Boontje N. M. Zaremba R. De Jong J. W. Janssen P. M. L.. (2003b). The effect of myosin light chain 2 dephosphorylation on Ca^2+^-sensitivity of force is enhanced in failing human hearts. Cardiovasc. Res. 57, 505–514. 10.1016/S0008-6363(02)00662-4 12566123

[B46] Van Der Velden J. Papp Z. Zaremba R. Boontje N. M. De Jong J. W. Owen V. J.. (2003c). Increased Ca^2+^-sensitivity of the contractile apparatus in end-stage human heart failure results from altered phosphorylation of contractile proteins. Cardiovasc. Res. 57, 37–47. 10.1016/S0008-6363(02)00606-5 12504812

[B47] Wang L. Muthu P. Szczesna-Cordary D. Kawai M. (2013a). Characterizations of myosin essential light chain's N-terminal truncation mutant Delta43 in transgenic mouse papillary muscles by using tension transients in response to sinusoidal length alterations. J. Muscle Res. Cell Motil. 34, 93–105. 10.1007/s10974-013-9337-x 23397074 PMC3656599

[B48] Wang L. Muthu P. Szczesna-Cordary D. Kawai M. (2013b). Diversity and similarity of motor function and cross-bridge kinetics in papillary muscles of transgenic mice carrying myosin regulatory light chain mutations D166V and R58Q. J. Mol. Cell. Cardiol. 62, 153–163. 10.1016/j.yjmcc.2013.05.012 23727233 PMC3809071

[B49] Wang Y. Xu Y. Kerrick W. G. L. Wang Y. Guzman G. Diaz-Perez Z.. (2006). Prolonged Ca^2+^ and force transients in myosin RLC transgenic mouse fibers expressing malignant and benign FHC mutations. J. Mol. Biol. 361, 286–299. 10.1016/j.jmb.2006.06.018 16837010

[B50] Weterman M. A. Barth P. G. Van Spaendonck-Zwarts K. Y. Aronica E. Poll-The B. T. Brouwer O. F.. (2013). Recessive MYL2 mutations cause infantile type I muscle fibre disease and cardiomyopathy. Brain 136, 282–293. 10.1093/brain/aws293 23365102

[B51] Yuan C. C. Muthu P. Kazmierczak K. Liang J. Huang W. Irving T. C.. (2015). Constitutive phosphorylation of cardiac myosin regulatory light chain prevents development of hypertrophic cardiomyopathy in mice. Proc. Natl. Acad. Sci. U.S.A. 112, E4138–E4146. 10.1073/pnas.1505819112 26124132 PMC4522794

[B52] Zhang Y. (2008). I-TASSER server for protein 3D structure prediction. BMC Bioinformatics 9:40. 10.1186/1471-2105-9-40 18215316 PMC2245901

